# Lost in translation? A qualitative study of representations and management of chronic depression in general practice

**DOI:** 10.1186/s12875-023-02019-3

**Published:** 2023-03-24

**Authors:** Audrey Linder, Daniel Widmer, Claire Fitoussi, Lucile Gagnebin, Yves de Roten, Jean-Nicolas Despland, Gilles Ambresin

**Affiliations:** 1 School of Health Sciences (HESAV), University of Applied Sciences and Arts Western Switzerland (HES-SO), Av. de Beaumont 21, 1011 Lausanne, Switzerland; 2 Department of Psychiatry, University Institute of Psychotherapy, Lausanne University Hospital (CHUV), Lausanne, Switzerland; 3 Department of Family Medicine, Faculty of Biology and Medicine, Unisanté, University of Lausanne, Lausanne, Switzerland

**Keywords:** Chronic depression, Focus Groups, General practitioners, Mental Health, Patient care

## Abstract

**Background:**

GPs are on the front line for the identification and management of chronic depression but not much is known of their representations and management of chronic depression.

**Objectives:**

To analyze GPs’ representations of chronic depression and to explore how they manage it.

**Methods:**

Three focus groups were conducted with 22 French-speaking general practitioners in Switzerland. The focus groups were transcribed and coded with MaxQDA. A detailed content analysis was carried out and the results were synthesized into a conceptual map.

**Results:**

GPs form representations of chronic depression at the intersection of expert and lay knowledge. When talking about patients suffering from chronic depression, GPs mention middle-aged women with complex psychosocial situations and somatic complaints. GPs’ management of chronic depression relies on the relationship with their patients, but also on taking care of them as a whole: psyche, body and social context. GPs often feel helpless and lonely when confronted with a patient with chronic depression. They insist on the importance of collaboration and supervision. As regards chronic depression management, GPs remain alone with patients suffering from complex biopsychosocial situations. In other situations, GPs seek the help of a psychiatrist, sometimes quickly, sometimes after a long approach. In each situation, GPs have to develop skills for translating patients’ complaints.

**Conclusion:**

GPs endorse a role of interpreter, making the physical presentation of their patient complaints move towards a psychological appreciation. Our results call for a renewed interest in GPs’ role as interpreters and the means to achieve it.

**Supplementary Information:**

The online version contains supplementary material available at 10.1186/s12875-023-02019-3.

## Background

Depression affects more than 200 million people worldwide [1]. After a first episode of depression, half of the people will have a further episode, “and in 10% it will follow a persistent or chronic course” [2]. However, international classifications are not unanimous on the definition of chronic depression (CD), which is a clinical entity at the intersection of different domains of mood disorders. It can be understood as (a) a major depressive episode that lasts, (b) a set of depressive symptoms that do not constitute a major depressive episode but are present almost every day, (c) repetitive major depressive episodes with no real remission between episodes, or (d) a combination of these elements. ICD-10 does not allow for a chronic course of major depressive disorder to be coded, while DSM-5 proposes the category of persistent depressive disorder. Therefore, we decided not to treat CD as an existing diagnosis, but rather to investigate what CD means for general practitioners (GPs), how do they recognize a patient suffering from CD and how they use the diagnosis of CD to organize treatment and possibly collaborate with psychiatrists^1^.

In fact, as far as depression is concerned, GPs are on the front line. In the UK, “all patients are registered with their own [GP], who provides almost all initial advice and management for illness”, which means that identification and management of depression is “a common experience for GPs” [3]. Similarly, a Swiss study showed that 96.9% of them treat patients with depression [4], and Swiss GPs estimate that around 30% of their patients suffer from depression [5]. One in two depressions treated by GPs is severe and recurrent [5] and around 73% of patients with severe depressive symptoms suffer from significant somatic problems [6]. Concurrently, there is a lack of guidelines for GPs on how to take care of chronic depression. In Switzerland, psychotherapy is reimbursed by social insurance and can be prescribed by any GP. It is, however, underprescribed [5].

Our article aims at answering two research questions: (1) what do GPs understand by the condition of “chronic depression”? (2) How do they manage chronic depression (treatment and referral)?

## Methods

### Study context

In Switzerland, out of a total of 38,500 practicing physicians in 2020, 20,300 practice in the ambulatory sector [7]. About 22% are qualified in general internal medicine, 4% are medical practitioners with a European title in general medicine, and 10% are psychiatrists [7]. Physicians with degrees in general internal medicine and medical practitioners are considered as GPs. The ambulatory system is liberal with remuneration based on the time devoted to the patient. Physicians can practice in an individual practice or in a group practice. GPs working in these settings usually practice independently and not as employees, while GPs practicing in outpatient clinics are generally employees. In private individual practices, the GP practices alone with minimal support staff and minimal technical resources. Technical and staff resources can vary considerably between group practices, depending on the number and specialization of physicians working together. Switzerland clearly lacks GPs in both urban and rural areas. The care of vulnerable populations is not a question of geographical area but linked more to the ability of doctors to take charge of complex situations.

### Population and sampling

Twenty-two participants were recruited by means of a mailing list of the GPs’ from the Vaud Society of Medicine, as well as through personal contacts (AL and CF during march 2017). Candidates answered voluntarily to the email they received or to the personal solicitation. AL was the person of contact for GPs interested in participating and responsible for the recruitment process. There was no refusal by personal contact. We used a purposeful variation sampling method, assuring diversity, considering age, gender, practice area and additional training in psychosocial and psychosomatic medicine (open to all MD in Switzerland) 2. Possibly GPs with this additional training have more experience in mental health. A consent form explaining the process of the study, the processing of the data, the remuneration, the rights and obligations as well as the qualities of the research staff was signed by every GP before their participation to the focus groups.

### Data collection

We conducted three focus groups with GPs in French-speaking Switzerland with respectively four, seven and eleven participants. Focus groups were conducted between January and March 2017 according to an interview guide (Table 1). Focus groups were led by a trained female sociologist (AL, PhD), along with two female undergraduate students in medicine (CF) and psychology (MH). One of the students (CF) co-animated the focus groups, while the other (MH) took notes to help the transcription. Focus groups took place in an outpatient clinic, in the city center of Lausanne, and their length was about one hour and a half each, without repetition. Since international classifications such as ICD-10 and DSM-5 do not share a common definition of chronic depression, focus groups appeared as more suited to capture a common definition of chronic depression emerging from participants’ private definitions, to enlarge participants’ profiles and to enrich answer contents. Given the diversity of definitions, we decided to anchor the discussions in the practical experience of the participants and started by asking GPs to individually write a clinical vignette describing the case of one of their patients suffering from CD. On the basis of these vignettes, we asked them to single out the characteristics of patients with CD. We then asked how they treat these patients and coordinate with psychiatrists^3^. Focus groups were audio-taped and transcribed verbatim by a professional. Socio-demographic and professional data such as gender, age, country of graduation, years since graduation, type of practice (individual/group/outpatient clinic), years since practice opening, localization (urban/semi-urban/rural) as well as whether they were specialized in family medicine or in psychosomatic medicine were collected.

**Table 1:** Interview guide

To begin this focus group, we suggest that you take a couple of minutes to think about a situation of chronic depression in your practice that you would like to describe, and write it down.
**1. Referring to the clinical situation you have just written, what do you think characterizes chronic depression in general practice?**
**2. How do you deal with a chronically depressed patient?** a. What type of support do you offer? b. Why would you offer this type of support? c. What difficulties do you encounter? d. How do you feel about these patients?
**3. In the case of chronic depression, how do you see the role of the psychiatrist?** a. Thinking about clinical situations that you know, can you tell us why and how you decide whether or not to call on a psychiatrist? b. How would your action be different and/or complementary to that of the psychiatrist in these situations? c. What should/could be done to make the collaboration between family physicians and psychiatrists go better?
**4. Has your representation of depression changed over the years?**

### Data analysis

Our theoretical framework was a grounded theory approach. The main research areas were defined by our research questions (representations of CD, management of CD, collaboration with psychiatrists). Codes were derived from the data through inductive open coding, in geronds or action verbs [8, 9]. The first focus group was coded individually by each member of the research team. After this was completed, the team established a joint draft of the coding list. The following focus groups were coded by three researchers – a sociologist (AL), a GP (DW) and a medical student (CF) – with the software MaxQDA. Each focus group was coded by two coders, who then proceeded to intercoding agreement. The potential disagreements were flagged by a “memo” and discussed in the team until an agreement was reached. Discussions also took place after coding the three focus groups, to proceed to the last agreements and delete or group together some of the sub-codes (axial coding, elaboration of categories and properties). At the end of the process, the new codes appeared to be slight variations of existing codes. Therefore, we considered that data saturation was sufficient. From the coded material, a detailed content analysis was carried out by the research group and a conceptual map was designed to synthesize our results. A session was organized for presentation of first results where the participants were invited.

## Results

The participants were fourteen men and eight women, aged between 30 and 72 years old, with a mean age of 51.8 years. They were experienced with a mean time since graduation of 25.0 years and since practice opening of 14.8 years. They worked either in an individual private practice (n = 10), a collective private practice (n = 7) or an outpatient clinic (n = 5), located in urban (n = 14), semi-urban (n = 6) or rural area (n = 2). About a half of them (n = 9) received a training in psychosocial and psychosomatic medicine. (Table 2)

**Table 2:** Participants socio-demographic characteristics and professional data

FG No	Age range	Gender M/F	Country of graduation	Years since graduation	Specialist in Family Medicine	Specialist in Psychosomatic Medicine	Type of practice	Years since practice opening	Localisation
1	40–44	M	Switzerland	19	Yes	Yes	Group (2–5)	10	Urban
1	50–54	F	Switzerland	26	Yes	Yes	Outpatient	16	Urban
1	55–59	F	Switzerland	32	Yes	Yes	Individual	17	Semi-urban
1	45–49	M	Switzerland	20	Yes	Yes	Group (2–5)	11	Rural
2	55–59	M	Switzerland	34	Yes	No	Individual	24	Urban
2	55–59	M	Switzerland	32	Yes	No	Group (> 5)	25	Urban
2	35–39	M	Spain	6	Yes	No	Outpatient.	-	Urban
2	65–69	M	Switzerland	40	Yes	No	Individual	26	Urban
2	35–39	M	Portugal	13	No	No	Outpatient	-	Urban
2	30–34	F	Switzerland	7	No	No	Outpatient	-	Urban
2	50–54	M	Switzerland	26	Yes	Yes	Group (2–5)	16	Semi-urban
3	40–44	F	Switzerland	15	Yes	Yes	Group (2–5)	4	Urban
3	70–74	M	Syria	47	Yes	No	Individual	27	Urban
3	60–64	F	Switzerland	36	Yes	No	Group (2–5)	25	Semi-urban
3	55–59	F	Switzerland	27	Yes	Yes	Individual	11	Semi-urban
3	40–44	F	Switzerland	19	Yes	Yes	Individual	10	Urban
3	30–34	F	Switzerland	6	No	No	Outpatient.	-	Urban
3	45–49	M	Switzerland	20	Yes	No	Individual	9	Urban
3	65–69	M	Switzerland	39	Yes	Yes	Individual	32	Rural
3	45–49	M	Switzerland	8	Yes	In training	Group (2–5)	1	Semi-urban
3	70–74	M	Switzerland	46	Yes	Yes	Individual	40	Semi-urban
3	60–64	M	Switzerland	31	No	No	Individual	21	Urban

*Note* Specialist in Psychosomatic Medicine = Member of the Swiss Association in Psychosomatic and Psychosocial Medicine; Outpatient = Public outpatient clinic

We present our results in three sections: characteristics of the patients treated by GPs, treatment and type of referral to psychiatrists.

### Characteristics of patients with chronic depression treated at the general practice

From the majority of GPs’ point of view, CD is not a precise diagnostic entity but a diagnostic nebula, consisting mainly of somatic complaints and complex psychosocial difficulties. It is similar to – and sometimes merges with – medically unexplained symptoms. Often, the patients themselves do not recognize the existence of a psychological problem.

When asked about the characteristics of patients with CD, GPs mainly talked about middle-aged women (45–65 years old). Their psychosocial situation is complex and characterized by precarious employment (nightshifts or irregular hours of work, work environment “at risk for anxiety and depression”); financial difficulties (debts, social insurance pension); isolation and loneliness; relationship problems with the family (drug or alcohol consumption, conflicts, domestic violence, divorce) but also at work (mobbing, dismissal); childhood trauma (mistreatment, sexual abuse) and, often, a history of migration, sometimes traumatic.

Somatic complaints are at the forefront of CD in general practice. Some GPs used the word “polyplaintive” to describe their patients with CD, stating that they always come at the GP’s with new physical complaints. Some GPs made the hypothesis that the somatic complaints were linked to the difficulty of thematizing the emotional problem: “I think that these people, the chronification – we’re talking about chronicity – it’s more like people we’re having trouble approaching. The people we could talk to about the problems, the real problems they have, either us or the psychiatrist, they will find solutions and I think we can no longer talk about CD” (FG2-GP8). According to some GPs, somatic complaints are due to the fact that “there’s something depressive about it, but it’s not said because there are no words to say it” (FG1-GP7), therefore it is expressed through “extreme pains (…) that often make them consult urgently, see a lot of specialists” (FG1-GP2).

Precise diagnosis was not considered of great importance, since GPs practice a “person-centered medicine” which focuses on the patient’s difficulties rather than on a specific pathology. Their representations of CD and of their patients mixed expert and lay knowledge. Some of them used the concept of “masked depression”, while others expressed the idea that patients with CD indulge in their suffering: “I have a lot of patients where (…) it seems that CD becomes a leitmotif in fact, that it is a label that suits them. In fact, it’s an operating mode” (FG3-GP2).

### Treatment of patients with chronic depression by general practitioners

Some patients are quickly referred to a psychiatrist, while others are treated in the long-term by GPs. For the latter, GPs mobilize a number of tools and resources. At the heart of their management of CD lies the relationship between their patients and themselves. GPs mentioned many times the importance of the bond, of trust and confidence. Some of them insisted that it is necessary to get away from the idea they have to *do* something, in order to actually *meet* the person. With experience, they learn to “prescribe themselves” as doctors, and one GP joked that their two main tools are “the stethoscope and the tissues”.

Some GPs emphasize the necessity of setting a framework for the consultation and seeing the patient regularly. For most patients, they try to elaborate on the somatic complaints, and for some, they prepare them for psychotherapy. Most participants insisted on the importance of global care in general practice, that is, to take care of the psyche as well as the body, but also to take care of the patient as a whole, taking into consideration the patient’s context. Some GPs try to highlight the patient’s resources and to activate them. Another set of tools mentioned by GPs are the prescriptions, such as sick-leaves, antidepressants or even complementary therapies (massages, sophrology, meditation, art or music therapy). Many GPs have received additional training (psychosomatic medicine, hypnosis, CBT, brief psychodynamic therapy) and use the acquired knowledge to help patients with CD.

GPs often feel helpless and lonely when confronted with a patient with CD:

Maybe the feeling of helplessness is also that feeling a bit distressing, when you think that these people, in the end we are really settled in chronicity, these people are clinging to us (…). We have to offer them this setting, this continuity, on the long-term, and sometimes we have the impression that nothing is moving, we don’t move forward. We know it is part of the game but despite everything, we are sometimes overtaken by this feeling of emptiness or lack of effectiveness. (FG1-GP7)

To face these feelings, most GPs insist on collaborating – in order to not be alone with the patient – and on taking care of themselves: “Our tools, at that moment, is to not get depressed ourselves, to hold up, so it is the supervisions, the intervisions, things like that” (FG1-GP2). GPs insisted on the importance of working with the healthcare network: “the tool is the network (…). I find that it’s very difficult to manage, but it’s still what makes it possible to hold up, because it’s really often that you have to hold for a long time, and alone it’s very ambitious!” (FG2-GP7). More generally, GPs expressed the need to “take care of the caregiver” in order to “avoid the compassion fatigue that exists in relation to these patients who are polyplaintive, who may not be very compliant with the treatment and who are not very positive” (FG2-GP3). Some GPs even linked up the caregiver’s condition with the patient’s: “the Balint groups are very useful. Because we can talk about it, and then we get better, we approach the patient differently, and the patient often gets better after we talk in the Balint group” (FG2-GP7). Finally, most GPs mentioned that treating CD becomes less difficult with experience and training, also because it gets easier to feel at ease with the idea that “it is ok” to “merely” take care of the patient if it is not possible to cure him/her.

**Fig. 1:**
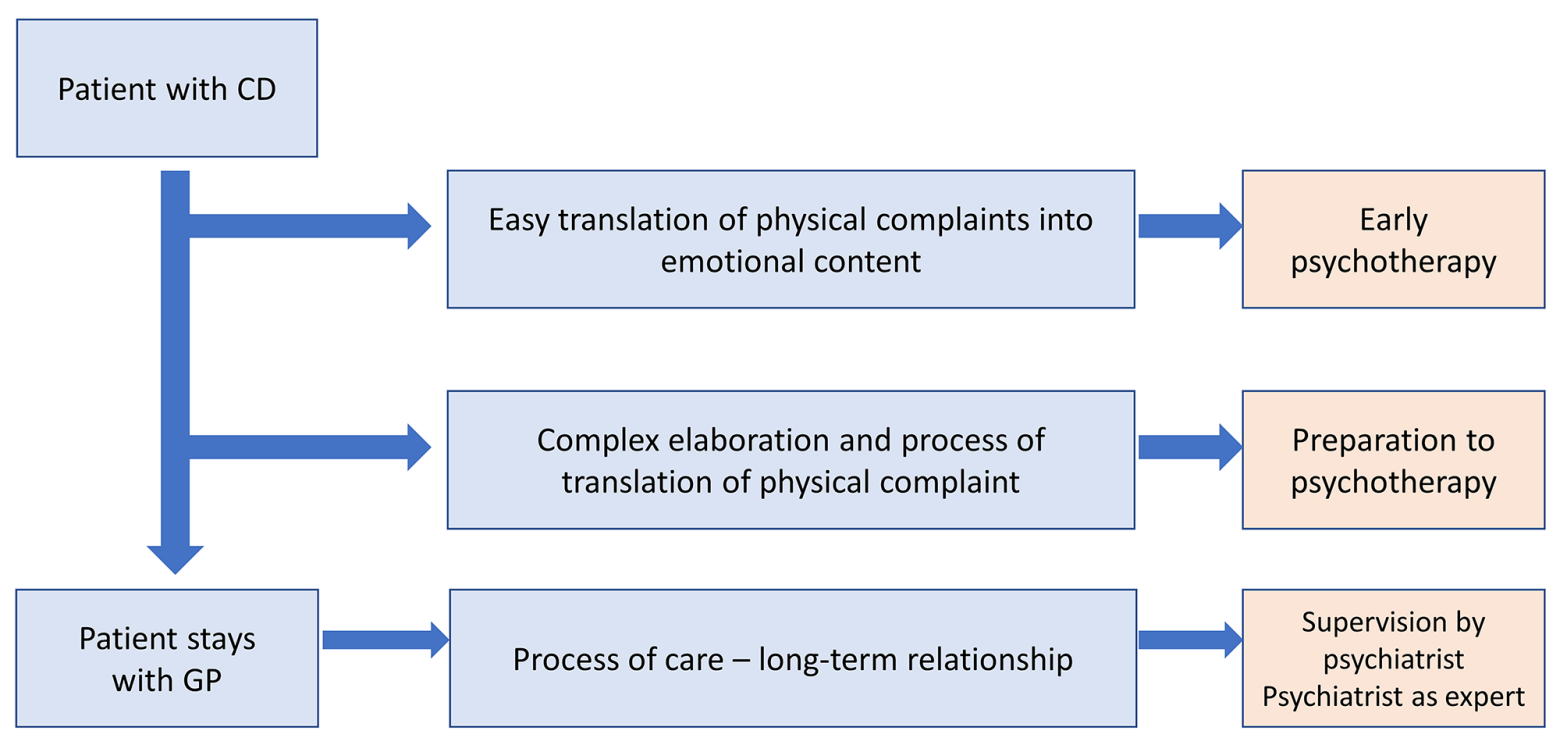
Three pathways for the patient with chronic depression in family practice

[Figure 1. Three pathways for the patient with chronic depression in family practice] *Three types of referral from general practitioners to psychiatrists*.

Regarding referral, most GPs consider three types of patients with CD (Fig. 1). The first type is called the “good patient for the psychiatrist” by some GPs. This type of patients are described as having good insight, being of the “intellectual type” and having a capacity to talk about their emotions. Such patients can be referred directly to a psychiatrist, either because they come to the GP with this request, or because the GP does not have enough time to see them regularly and suggests seeing a psychiatrist. According to GPs, these patients are the ones who accept the referral more easily (FG1):

**GP7:** I have the feeling that we have to select the patients, and send the “good cases” – in quotation marks – to the psychiatrist. (…)

**Interviewer:** What characterizes the “good cases” for the psychiatrist?

**GP7:** For me, they are people who are able to read their emotions, to elaborate, to connect things, it’s the intellectual profile, if you wish. I would tend to refer these patients to the psychiatrist.

**GP2:** That is, they will accept to go easily, too! (…) Because others can be offered to go, but they may not understand.

The second type concerns patients who need to be prepared by the GPs before they can be referred. GPs’ work is to help them put words on their emotions and link them with their physical pains. Temporality is a major component of the evolution: “it takes years before, gradually, the emotional material emerges, and then we can start to bring things out, through the relationship” (FG1-GP7) says one GP, adding that there are cases “where we feel them ripe, like a ripe fruit, to make an introspective approach. At that point, I have the impression that when they are really interested in digging, in entering psychotherapy, (…) you have to seize that moment to guide them to the psychiatrist”.

Finally, the third type of patients present their depression mainly through somatic complaints, which they cannot relate to depression or emotions. This type of patients is the one GPs talked the most about, since these are usually not referred to a psychiatrist and are long-term patients in the GPs’ consultation. However, they might benefit from the psychiatrist in different ways. First, when conflicts arise with insurances while they are on a sick leave or have been getting a benefit from health insurance for a long time. In this case, psychiatrists join in the patient’s treatment for strategic reasons, since GPs have noticed that psychiatrists’ expertise is better recognized by insurances than GPs’. Psychiatrists’ specific expertise is also required when GPs struggle with patient’s medication. Another situation in which these patients end up being treated by a psychiatrist is when they attempt to commit suicide – or threaten to – and are admitted in a psychiatric hospital. Finally, in some situations the patients benefit from the psychiatrist in an indirect way, when GPs ask for a supervision, usually when they have difficulties treating the patient and feel helpless.

## Discussion

### Summary

Our study aimed to analyze GPs’ representations and management of chronic depression. To this end, we carried out a detailed content analysis of data collected via three focus groups bringing together 22 GPs. Our study found that: (1) GPs form representations of chronic depression at the intersection of expert and lay knowledge; (2) GPs’ management of chronic depression relies on the relationship with their patients as well as on taking care of themselves, and; (3) whether GPs manage chronic depression alone or whether they seek the help of a psychiatrist, they have to develop skills in translating patients’ complaints. Results were synthesized into a conceptual map displaying 3 types of referral to the psychiatrist. We further discuss the findings below.

### Context with other literature

#### Diagnosis of chronic depression by GPs as process and pattern

Diagnosis is both a category – “a list of disease” – and a process – “the thing that the physician does: the conclusion reached, or the act of coming to that conclusion” [10]. The role of diagnosis is to organize illness, by “identifying treatment options, predicting outcomes, and providing an explanatory framework” [11]. By describing the 3 different pathways of care GPs emphasize the processual dimension of diagnosis. However, CD is not a categorial diagnosis *per se*. For our research participants, CD is a clinical entity at the intersection not only of different domains of mood disorders but also of physical and psychosocial disorders. Our research shows that GPs form their own representations of CD – and of patients suffering from CD – in order to be able to identify it and organize its treatment. These representations intervene in the process of “pattern recognition”, which consists in “rapid non-analytical matching of clinical presentation with a pattern previously formed of constructs of clinical signs and symptoms (or pattern) in memory”, the retrieval of this pattern being “triggered by recognition of key features within the case” [12].

#### Patients’ (un)speakable emotions and ability to grasp cues and concerns

In our study, one key feature is the patient’s (in)ability to talk about their emotions or psychological distress. The role of human speech is important in its ability to express one’s inner world [13]. The speech activity seeks to capture what is happening in the patient’s intimate realm when it manifests itself as a somatic complaint. This intimate realm colors the patient’s speech and GPs are sensitive to it. GPs probably recognize in their patient’s speech, more or less consciously, that these are points of contact between the patient’s explicit discourse and what they cannot yet say. In GPs representations, these points of contact must be grasped at the good moment and open the way to psychotherapy. This feature seems to belong more to the ‘gut feeling’ than to objective criteria of depression [14]. Consistent with our findings, the importance of the conversation between the GP and the patient to break the cycle of failure to achieve remission and address the possibility of psychological interventions in the treatment of depression has been recently highlighted in a systematic review and thematic synthesis of the experiences of treatment-resistant mental health condition in primary care [15].

Research participants value the long-term relationship that gradually allows the psychological work of their patients. The relationship is made of “stethoscope and tissues”: tissues as an indicator of their quality of presence and ability to touch something emotional in their patients, stethoscope as a reminder to take care of the somatic complaint. This GP emotional literacy is based both on their awareness of what is going on, on communication competencies to underline cues and concerns from the patient, on and their ability to translate it into words. This is the work of the interpreter who makes the physical presentation move towards a psychic appreciation [13, 16, 17].

#### GPs’ psychotherapic work: translating somatic complaints into words and emotions

This question concerns the status of psychotherapy in general medicine that Enid Balint started to explore when she set for “enabling doctors to recognize and understand their patients’ complaints (…) in terms of personal conflicts and problems, and then to use this understanding so that it should have a therapeutic effect” [18]. Psychotherapeutic interventions in primary care are effective, but are uncommonly delivered by GPs [19]. However, GPs in our study unequivocally stated that, as interpreters, they fostered a psychological work in their patients. Our results call for a renewed interest in GPs’ role as interpreters and the means to achieve it.

Referral to the psychotherapist rests on the GP’s capacity to utter the right words, and foster the emergence of words on the intimate realm in their patients. However, when confronted with a patient who uses somatic symptoms to express the CD they suffer from, GPs can also be struck by speech impotence. Without the capacity of translation, GPs can struggle to share the diagnosis with their patient. Our findings are in line with a previous study exploring the decision process to refer to specialists in tertiary healthcare in Switzerland [20]. Tzartzas et al. [20] showed that GPs in training at an outpatient clinic refer their patients to various specialists for problems that cannot be explained only in biomedical terms but also for reasons related to emotionally charged interactions and relationships with patients. Both study findings indicate the importance of considering these issues when referring to a specialist. Our study adds to Tzartzas et al. [20] findings in indicating the importance of a preliminary psychotherapeutic work done by GPs when considering referral to a psychiatrist. In other words, working through the decision to refer with the patient in the relationship seems of particular importance to GPs considering referral to the psychiatrist of a patient with chronic depression.

As our results suggest, tools help GPs bring their patient to the psychotherapist who pursues the translation endeavor, eventually offering them some sort of Rosetta stone^4^. When translation or referral to the psychotherapist is not possible, GPs turn to the psychiatrist as supervisor, or towards taking care instead of curing. Recent studies also indicate that GPs may turn towards social prescription [21], or lean on a theoretical explanation to withstand the situation [22].

### Strengths and limitations

The main strength of the study is the inclusion of both younger GPs working in outpatient clinics and more experienced GPs working in their private practices, allowing the interaction between two generations of GPs and to provide a large description of patients with CD and the management of CD. Furthermore, at the analysis level, the research team included a sociologist, a GP, and a psychiatrist ensuring that the topics covered in the interviews were considered from perspectives relevant to the objective of the study. Unfortunately, our study is limited in generalizability by the specificities of the Swiss medical system. As GPs are paid according to the time devoted to the patient, they may take more time for the conversation with CD patients to discuss the understanding and management of CD and time may foster an enhanced translation of issues particular to CD. Translation, and their related skills, takes time.

### Implications for research and practice

As regards clinical care, our results converge on the idea of the need to translate patients’ complaints. GPs are encouraged to develop tools helping them with the translation process, alongside their relational skills and attitudes. Results of the study indicated that in parallel with tools stemming from the usual framework of general practice, taking care of themselves and sharing with colleagues (supervisions, Balint groups) were particularly helpful when treating patients with CD. Supervisions and intervisions were important to GPs, because they got to feel their patients’ loneliness and distress to the point of experiencing it. It is therefore important to be able to speak about it in Balint groups: discussing a difficult situation helps GPs change their attitude and find an appropriate way to continue to care for their patient.

As regards research implications, collaborating with a sociologist promoted the focus on the actual clinical experiences and processes for the diagnosis and care of CD.

## Conclusion

The question of translation in general medicine underlines the hermeneutical dimension of this profession, which should always balance the algorithmic thinking. Clinical situations where translation seems impossible and which constitute a hermeneutical void deserve full attention to foster patients’ access to the full span of CD care.

## Electronic supplementary material

Below is the link to the electronic supplementary material.

Supplementary Material 1

## Data Availability

The data underlying this article cannot be shared publicly for the privacy of individuals that participated in the study. The data will be shared on reasonable request to the corresponding author.

## Footnotes

1 In Switzerland, psychiatrists are also trained in psychotherapy, so they have the dual title of psychiatrist-psychotherapist.

2 In Switzerland, GPs have the opportunity to follow an additional training in psychosomatic and psychosocial medicine, which is recognised by the professional association.

3 Due to space limitations, we have decided not to address the issue of coordination and collaboration between GPs and psychiatrists in this article.

4 The Rosetta Stone is what helped scholars crack the code of hieroglyphics, the ancient Egyptian writing system.

## Abbreviations

CD: Chronic Depression
FG: Focus Group
GP: General Practitioners
